# The adaptation of The parental reflective functioning questionnaire adolescent version to the Hungarian language and presentation of its psychometric characteristics

**DOI:** 10.1192/j.eurpsy.2022.543

**Published:** 2022-09-01

**Authors:** B. Szabó, M. Miklósi, M. Boda, J. Futó

**Affiliations:** Eötvös Loránd University, Department Of Developmental And Clinical Child Psychology, Budapest, Hungary

**Keywords:** mentalization, developmental psychology, reflective function, parental reflective function

## Abstract

**Introduction:**

Parental reflective function is the ability of a parent to attribute mental states to their child and to themselves. The Parental Reflective Functioning Questionnaire is widely used for the measurement of this construct, the adolescent version of which can be used by parents of children aged 12-18.

**Objectives:**

The aim of our research was to adapt the adolescent version of The Parental Reflective Functioning Questionnaire to the Hungarian language.

**Methods:**

In our study 240 mothers completed the adolescent version of The Parental Reflective Functioning Questionnaire (PRFQ-A), and the Reflective Function Questionnaire (RFQ).

**Results:**

Confirmatory factor analysis did not confirm the original three-factor structure. The principal component analysis resulted in a two-factor structure. Factors corresponded to the original questionnaire’s certainty in mental states (Alpha = .81) and interest and curiosity subscales (Alpha = .70). When analyzing the relationship between parental reflective function and reflective function, the subscales of the parental reflective function questionnaire were examined with two types of median coding in addition to polar coding. During the first median coding, the frequency of scores in the middle of the scales reflected optimal mentalization, while the frequency of extreme values on the scales corresponded to less favorable reflective functioning. With the second median coding, hypermentalization and hypomentalization subscales were also created. The second median transcoding proved to be the most suitable for capturing the relationship between RFQ and PRFQ-A.

**Conclusions:**

The questionnaire proved to be a reliable measure on the Hungarian sample and we recommend using the additional subscales.

**Disclosure:**

No significant relationships.

